# Effect of an individually tailored one-year energy balance programme on body weight, body composition and lifestyle in recent retirees: a cluster randomised controlled trial

**DOI:** 10.1186/1471-2458-10-110

**Published:** 2010-03-05

**Authors:** Andrea Werkman, Paul JM Hulshof, Annette Stafleu, Stef PJ Kremers, Frans J Kok, Evert G Schouten, Albertine J Schuit

**Affiliations:** 1 Wageningen University, Division of Human Nutrition, PO Box 8129, Internal code 62, 6700 EV Wageningen, The Netherlands; 2 TNO Quality of Life, Physiological Sciences, PO Box 360, 3700 AJ Zeist, The Netherlands; 3 Maastricht University, Department of Health Education and Promotion, PO Box 616, 6200 MD Maastricht, The Netherlands; 4 National Institute for Public Health and the Environment, PO Box 1,3720 BA Bilthoven, The Netherlands; 5 Vu University, Faculty of Earth and Life Sciences, Department of Health Sciences, De Boelelaan 1085, 1081 HV Amsterdam, The Netherlands

## Abstract

**Background:**

The increased prevalence of overweight and obesity warrants preventive actions, particularly among people in transitional stages associated with lifestyle changes, such as occupational retirement. The purpose is to investigate the effect of a one year low-intensity computer-tailored energy balance programme among recent retirees on waist circumference, body weight and body composition, blood pressure, physical activity and dietary intake.

**Methods:**

A randomised controlled trial was conducted among recent retirees (N = 413; mean age 59.5 years). Outcome measures were assessed using anthropometry, bio-impedance, blood pressure measurement and questionnaires.

**Results:**

Waist circumference, body weight and blood pressure decreased significantly in men of the intervention and control group, but no significant between-group-differences were observed at 12 or at 24-months follow-up. A significant effect of the programme was only observed on waist circumference (-1.56 cm (95%CI: -2.91 to -0.21)) at 12 month follow up among men with low education (n = 85). Physical activity and dietary behaviours improved in both the intervention and control group during the intervention period. Although, these behaviours changed more favourably in the intervention group, these between-group-differences were not statistically significant.

**Conclusions:**

The multifaceted computer-tailored programme for recent retirees did not appear to be effective. Apparently the transition to occupational retirement and/or participation in the study had a greater impact than the intervention programme.

**Trial registration:**

Clinical Trials NCT00122213.

## Background

The increasing prevalence of overweight and obesity also affects the older population [1] and prevention of weight gain is also important in this population [2,3]. Weight gain is more common during transitional stages [3], such as occupational retirement at the sixth decade. Changes in physical activity and dietary behaviour, possibly induced by the retirement, contribute to this phenomenon. Moreover, with biological ageing, fat mass (mostly abdominal fat mass) increases and fat-free mass (mostly muscle mass) decreases. However, the extent differs between men and women and does not necessarily coincide with weight gain [1,4,5]. Abdominal obesity is associated with increased risk for cardiovascular diseases, diabetes mellitus type II and other chronic diseases [6]. Hence, the period of occupational retirement is a good moment to intervene, because it may lead to changes in diet and physical activity and consequently lead to weight gain and abdominal obesity [7-9].

Ideally, a behavioural intervention for this purpose should aim for small though sustainable changes to prevent gradual weight gain [10]. Preferably one should be physically active for 45-60 minutes per day [11] and consume a low energy-density diet in appropriate portion sizes that is rich in dietary fibre [12]. To increase the feasibility of large scale dissemination it is designed to be accessible for a large amount of the population at low costs [7].

We developed and evaluated a one-year multifaceted programme including these factors using computer tailored feedback on physical activity and diet [13]. The programme was evaluated for immediate and long term sustainability effects among a group of recent retirees. We hypothesised that the intervention group would maintain its body composition and physical activity and dietary behaviour in the years following occupational retirement, whereas the control group would show an average increase in waist circumference of 0.5 cm per year [13]. Measurements were taken at 12 and 24 months follow-up and included the primary endpoints waist circumference and body weight. We also measured other anthropometrics to evaluate body composition changes, blood pressure as indicator of general health status, physical activity behaviour and dietary intake. Secondary analyses were performed in order to evaluate whether the programme would be more effective in pre-defined subgroups based on socio-demographic characteristics [14].

## Methods

### Participants and recruitment

**Figure 1:**
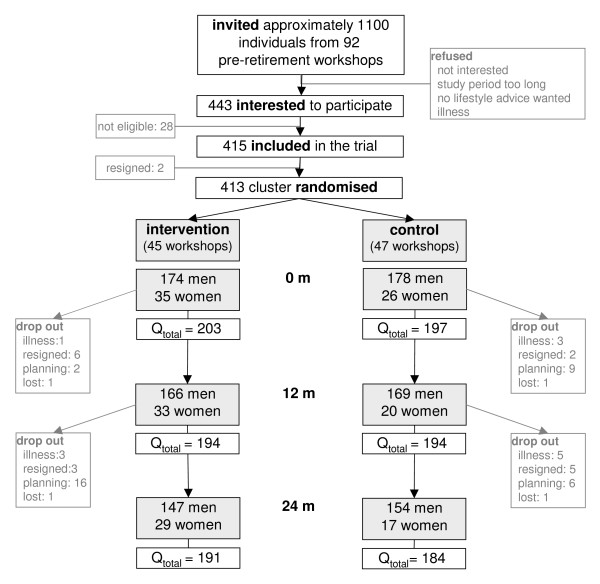
**Flowchart of all participants in the WAAG-Study**. Please note that all participants included in the WAAG-Study are included in this flowchart. Results of the intervention effectiveness are only presented for men, because of low numbers of women.

Subjects were eligible for participation in the WAAG-Study (Wageningen Approach against fat Accumulation and weight Gain) if they were recent retirees (date of retirement maximum six months before or after baseline measurement), aged 55-65 years, and not undergoing any medical treatment that might affect body composition. Participants were recruited from pre-retirement workshops as offered by employers to approximately 10% of the Dutch retiring population. During such a five-day workshop several topics are discussed in order to prepare retirees for the new phase in life, e.g. changes in the household after retirement, health and vitality. Workshops were held all across The Netherlands. Approximately 1,100 workshop attendees were invited to participate in the WAAG-Study from September 2003 to mid March 2004. First follow-up measurements were conducted from September 2004 to the end of February 2005, and final follow-up measurements were conducted from September 2005 to the end of February 2006. The Medical Ethics Committee of Wageningen University approved the study protocol and all participants gave written informed consent upon enrolment after they received written and verbal information about the trial [13]. In total 443 persons expressed their interest of which 415 were eligible and included (Figure 1).

### Study design

All persons attending one single workshop (cluster) who were willing to participate were physically examined at the location of the workshop. Furthermore, questionnaires on demographics, physical activity, dietary intake and psychosocial determinants were handed out. Within one week after the workshop the clusters were randomly allocated to either the intervention or the control group. Cluster randomisation was performed in order to avoid induction of favourable behaviour change of individuals in the control group through their contacts with fellow participants in the intervention group. A randomisation list was generated beforehand by an independent person and took into account the number participants per workshop and the number of included clusters per week. Due to the nature of the study it was not blinded.

Follow-up physical examinations were scheduled 12 ± 0.5 months (on average 11.9 ± 0.5 (mean ± SD); range 10.0-14.8 months) and 24 ± 1.0 months (on average 23.6 ± 0.9; range 21.4-26.7 months) after the baseline examinations. At 12 months follow-up 94% and at 24 months follow-up 84% of the participants returned for re-examination. Drop out was mostly due to planning problems, since not all participants could be scheduled for an appointment within the set limits. There were no differences between those who dropped out (n = 25 after the first year; n = 66 after the second year) and those who remained.

### Energy balance intervention programme

The intervention programme [13] was developed according to the Intervention Mapping protocol [15]. Prerequisites were that the programme was easy to implement and took into account individual preferences. It further aimed at small and sustained adaptations in physical activity and/or diet.

#### Intervention group

**Figure 2:**
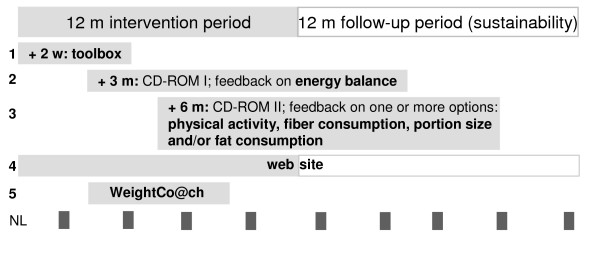
**Overview of the one-year intervention programme**. Note: +2w = 2 weeks from baseline, +3 of +6 m = 3 or 6 months from baseline. Solid bars represent intervention modules that were sent to the intervention group over the course of the 12 m intervention period. No additional information related to diet, exercise or a healthy weight was provided between 12 m - 24 m follow-up period. Both intervention and control group received general newsletters (NL) to increase compliance at 24 m follow up.

Five programme modules were provided to participants of the intervention group during the one year intervention period as shown in Figure 2. Participants could freely choose to make use of the modules or not. Modules 1 and 2 aimed to increase awareness of the energy balance concept and module 3 aimed to improve dietary and/or physical activity behaviour. Module 1 (sent within two weeks after the baseline measurement) was provided as a toolbox and included an information leaflet and several energy balance tools, e.g. a pedometer and a waist tape. Module 2 (sent 3 months after baseline) was a CD-ROM providing individually computer-tailored feedback on BMI, its health consequences and energy balance behaviour. In module 3 participants could receive computer-tailored feedback regarding: physical activity, fibre consumption, portion sizes of energy dense foods and fat consumption. This module was sent 6 months after baseline. Participants without access to a computer (n = 22) were interviewed (AW) and received printed feedback by mail. Modules 4 and 5 were accessible via the study website which was available during the two-year study period. After login, participants could find more information about diet and physical activity behaviour, participate in a forum and use links to other websites (module 4). Module 5 was an interactive weight maintenance programme (Weight Co@ch [16]) that provided a written tailored advice based on reported body weight, a food frequency questionnaire and a physical activity questionnaire [16]. Finally, the intervention group received newsletters every 2-3 months that contained study information, information about diet and physical activity and encouragements to use the modules [13].

#### Control group

During the total study period of two years, the control group was provided with newsletters with general information about the study, such as study progress, and information about art exhibitions and city trips for instance. They could not login to the website and had access to the general information about the study design only [13].

### Outcome measurements

Baseline physical examinations were performed at the site of the pre-retirement workshop, between 11 am and 2 pm. Follow-up examinations were conducted at various community health centres across the Netherlands at the same time of day. Examinations were carried out by the same two trained researchers over the total study period. Most participants (76%) were examined by the same researcher at baseline and 12 months follow-up and at the 24 months follow-up one researcher performed all examinations. Physical activity, diet, demographic and utilisation information were assessed by questionnaire. Questionnaires were handed out (baseline) and sent by mail (follow-up measurements) and were either returned in pre-paid envelopes (baseline) or handed in at follow-up physical examinations (12 and 24 months).

#### Demographic data

Date of birth, date of retirement, physical activity level of their last job, and educational level were assessed by questionnaire. Activity level of the former job was based on four types of activities and ranged from non-active, e.g. administrative job, to very active, e.g. postmen. Highest attained education was categorised as 'low' (primary school or lower vocational education), 'medium' (high school or medium vocational education) or 'high' (higher vocational education or university degree).

#### Utilisation data

We also collected information on the use of the different modules of the intervention. Participants self-reported at all follow-up measurements whether or not they have used the modules once, twice or more. This is stated as utilisation per module and presented as a proportion of participants that returned the questionnaire.

#### Anthropometry

All anthropometrical measurements were performed while participants wore underwear only. Body weight was measured to the nearest 0.2 kg after regular calibration and height to the nearest 0.1 cm (weight by SECA 888 and height by SECA 225; Vogel&Halke GmbH, Hamburg, Germany). Circumferences of the upper-arm, waist, hip, thigh, and calf were measured twice to the nearest 0.1 cm with a non-stretchable plastic measuring tape on the non-dominant side of the body, according to standard protocol [17,18]. Abdominal sagittal diameter was measured twice with participants in a supine position using a Holtain-Kahn abdominal caliper (Holtain Ltd., UK) [19].

#### Body fat

A single frequency (100 kHz), tetra polar, body impedance analyser (BCM Controller, Data Input, Frankfurt, Germany) was used to estimate total body water according to TBW (kg) = 2.896 + 0.366*HEIGHT^2^(cm)/RESISTANCE_100 kHz _+ 0.137*WEIGHT(KG) + 2.485*SEX (1 = men; 0 = women) [20]. From total body water, we calculated percentage total body fat as 100%*((WEIGHT -TBW)/0.732)/WEIGHT[21].

#### Blood pressure

Blood pressure was assessed with participants in supine position using the average of two standard automatic blood pressure measurements (BOSO Oscillomat 751, Bosch&Sohn, Jungingen, Germany).

#### Physical activity

Changes in physical activity were assessed with the validated Dutch version of the Physical Activity Scale for the Elderly (PASE) [22]. This brief questionnaire is designed to assess physical activity of the previous week in older people, aged 65-100 years, and is sensitive of detecting small changes over a short period of time. PASE scores range from 0 to 400, higher scores indicating greater activity levels [23,24]. To calculate the PASE score the frequency and duration of the following activities was assessed: walking outside and bicycling; light intensity recreational activity; moderate intensity recreational activity; high intensity recreational activity and muscle strengthening activities. Frequency was recorded as never (0), seldom (1-2 days), sometimes (3-4 days) and often (5-7 days). Duration was categorised as 'less than 30 minutes' 'between 30 minutes and 1 hour' (from the 'less than 1 hour' category in the original version), '1-2 hours', '2-4 hours', or 'more than 4 hours'. Performance of six household activities (light and heavy housework, home repair, lawn work, gardening and taking care of others) were recorded as yes (1) or no (0) and summed (total score ranged from 0-6). Work (paid or voluntary) related activities were recorded in total hours/week and classified into four categories of intensity of physical activity during work.

Total PASE score was computed by multiplying the amount of time spent per activity (hours/day) and participation in the household activities by the item weights and then summing these products. The item weights indicate the contribution of each item to the overall PASE score; highest weights were assigned to the more strenuous types of activities [23]. Six questionnaires were excluded because more than half of the 11 items for the PASE score were missing. If less than 6 questions were missing, assumptions were made to estimate PASE score. If frequency was missing and duration was known, frequency was set at '1-2 days per week'; if duration was missing and frequency was known, duration was set at ' less than 30 minutes'; if frequency and duration were missing, the score was set at 0. If household activities were missing they were set at 0.

Further, we derived total time (min/week) spent on bicycling and walking as an indicator for routine daily activities and total time (min/week) spent on moderate and high intensity and muscle strengthening activities as an indicator of recreational and sports activities.

#### Dietary intake

Changes in the diet were assessed with a validated, semi-quantitative food frequency questionnaire (FFQ). The FFQ has been developed to estimate intake of fat, fatty acids, cholesterol and energy in adults using a reference period of four (habitual) weeks [25]. All FFQs were checked by a dietician and if necessary participants were contacted to collect additional or missing information.

Fruit intake (g) was calculated by summing the amounts of fresh fruits. Vegetable intake (g) was calculated by summing the amounts of cooked, fried and raw vegetables. The sum of fruit and vegetable intake (g) was adjusted for energy intake (MJ). Furthermore, total fat intake (en%) and total energy intake (MJ/day) were derived for the FFQ.

To estimate portion size, we used the total number of servings (of standard portion sizes) per month of certain energy dense products that are frequently consumed in the Netherlands: sliced meat, meat, beer and wine. To illustrate small adaptations, the number of sugar cubes in cups of coffee and tea, and milk added to a cup of coffee were evaluated.

Subjects reporting extreme differences between baseline and 12 months follow-up (60% increase or decrease in energy intake (MJ/day)) were excluded from the analyses (n = 6).

### Statistical analyses

We hypothesised that waist circumference in the control group would increase by 0.5 cm (standard deviation of difference = 1.3 cm) per year and that it would remain stable in the intervention group [13]. Based on these data the sample size for the WAAG-Study was calculated. To address the cluster randomisation we included 20% more individuals (design effect = 1.2) [26,27] and to control for expected drop out 25% was added to the sample size. Thus, at least 200 participants per group were needed to observe significant differences between the two groups at the 5% confidence level with 80% statistical power.

The effect estimates of the intervention included all participants who had provided at least one follow-up measurement. Data were analysed using mixed models with a random cluster effect allowing each cluster to have its own intercept. Analyses were performed using SAS (proc mixed; SAS for Windows, SAS Institute Inc. Cary, NC, USA; version 9.1). Models were constructed with the follow-up measurements as dependent variable and the baseline measurement as covariate. The estimate for treatment effect reflects the between-group-difference at follow-up, corrected for the value at baseline.

Additionally, we calculated effect sizes, a frequently used measure to demonstrate the magnitude of the effect of an intervention programme. The standardised effect size is calculated as the difference between the mean changes between the intervention and control group divided by the pooled standard deviation [28]. Effect sizes (Cohen's *d*) were interpreted according to the Cohen's guidelines and indicate the size of the effect of the programme. Cut off points for small effects are *d *< 0.32, medium effects as 0.33 <*d *< 0.55 and large as *d *> 0.56 [28]. We also performed per protocol analyses for waist circumference and body weight in order to test whether the utilisation of the key modules (modules 2 and 3) affected the outcomes at 12 and 24 months from baseline. We compared the users in the intervention group with a randomly selected sex-matched group of controls.

Secondary analyses were performed for waist circumference and body weight and fat intake and energy intake at 12 months follow-up in pre-defined subgroups of participants having low educational level (primary school only or lower vocational education), physically active former job, a BMI ≥ 30 kg/m^2 ^and a waist circumference ≥ 102 cm [29]. Also analyses were performed to investigate if utilisation of the intervention might influence the outcomes.

All statistical analyses were performed for men only, because age related changes in body composition differ between sexes and the number of women was too small to have sufficient power to draw conclusions. Statistical significance was set at p < 0.05 for all tests performed.

## Results

By cluster randomisation, 174 male participants were assigned to the intervention group and 178 men to the control group (Figure 1). They were on average 59.5 years old (Table 1) and had a BMI of approximately 27 kg/m^2 ^(Table 2). The toolbox (module 1) was used by 82% of the group, the first CD-ROM (module 2) by 72% and the second CD-ROM (module 3) by 41% of the group. The exposure to the website (module 4) and its interactive component (Weight Co@ch (module 5)) was lower, 54% and 16% respectively.

**Table 1:** Characteristics of the study population at baseline

	intervention	control
Number	174	178
Age (years)	59.5 ± 2.5	59.4 ± 2.3
Energy intake (MJ/day)	9.5 ± 2.1	9.8 ± 2.3
Total fat intake (en%)	34.6 ± 5.4	33.6 ± 5.3
Physical activity (PASE^a^)	116 ± 65	122 ± 68
Compliance (%) with norm for		
*Physical activity (> 30 min > 5 day/week)*	62	60
*Fruit and vegetable norm (> 350 g/day)*	30	35
*Fat intake (< 35 en%)*	61	60
Low educational level (%)	25	23
Current smokers (%)	9	12
Living alone (%)	7	2*
Active latest job (%)	35	43
Perceived health (%)		
*Excellent + very good*	32	28
*Good*	60	67
*Bad*	8	5
Hypertension drugs (%)	14	18
Cholesterol-reducing drugs (%)	9	14
Diabetes mellitus (%)	4	2

Values are presented as mean ± standard deviation or as percentages. Note that questionnaire derived data may have missing values; ^a ^Physical Activity Scale for the Elderly (range 0 (non active) - 400 (very active)); * p for association < 0.05 (χ^2^).

**Table 2:** Change and treatment effect for body composition in men after A) 12 and B) 24 months follow-up

A)	Baseline		Follow-up at 12 months			
			Change		Treatment effect^†^	*d*^‡^
	intervention	control	intervention	control		
Number	174	178	166	169		
WC^a ^(cm)	99.2 ± 9.5	100.4 ± 9.2	-2.32 ± 3.24**	-1.9 ± 3.06**	-0.48 (-1.16;0.2)	-0.13
BW^a ^(kg)	85.1 ± 11.9	86.1 ± 11.4	-1.86 ± 3.08**	-1.62 ± 3.03**	-0.28 (-0.97;0.42)	-0.08
BMI^a ^(kg/m^2^)	26.7 ± 3.6	27.3 ± 3.1	-0.49 ± 1.01**	-0.43 ± 0.98**	-0.07 (-0.29;0.15)	-0.06
HC^a ^(cm)	101.9 ± 5.4	102.2 ± 5.3	-1.74 ± 1.70**	-1.53 ± 1.88**	-0.24 (-0.66;0.17)	-0.12
TC^a ^(cm)	56.8 ± 3.6	57.0 ± 3.3	-1.32 ± 1.49**	-1.15 ± 1.76**	-0.17 (-0.55;0.22)	-0.10
SagD^a ^(cm)	21.8 ± 2.7	22.3 ± 3.0	-1.07 ± 1.42**	-1.17 ± 1.77**	-0.01 (-0.33;0.31)	0.06
CC^a ^(cm)	38.8 ± 2.7	39.1 ± 2.3	-0.93 ± 1.08**	-0.85 ± 0.81**	-0.09 (-0.30;0.12)	-0.08
AC^a ^(cm)	32.6 ± 2.6	32.8 ± 2.5	-0.73 ± 1.15**	-0.59 ± 1.01**	-0.15 (-0.38;0.08)	-0.13
SBP^a ^(mmHg)	142.7 ± 16.8	145.6 ± 17.9	-6.50 ± 9.93**	-4.59 ± 12.45	-2.36 (-4.72;0.01)	-0.17
DBP^a ^(mmHg)	86.1 ± 10.1	86.1 ± 8.9	-4.03 ± 6.62**	-2.79 ± 7.23**	-1.25 (-2.66;0.15)	-0.18
TBF^a ^(%)	30.4 ± 4.5	30.6 ± 4.7	-0.26 ± 2.23	-0.31 ± 4.13	0.02 (-0.68;0.72)	0.02
						
**B)**	**Baseline**		**Follow-up at 24 months**			
			**Change**		**Treatment effect**^†^	
	**intervention**	**control**	**intervention**	**control**		
Number	174	178	147	154		
WC^a ^(cm)	99.2 ± 9.5	100.4 ± 9.2	-1.06 ± 3.48**	-1.08 ± 3.60**	-0.10 (-0.82;0.79)	0.01
BW^a ^(kg)	85.1 ± 11.9	86.1 ± 11.4	-1.47 ± 3.66**	-1.58 ± 3.96*	0.10 (-0.77;0.97)	0.03
BMI^a ^(kg/m^2^)	26.7 ± 3.6	27.3 ± 3.1	-0.37 ± 1.12**	-0.40 ± 1.29*	0.02 (-0.27;0.30)	0.02
HC^a ^(cm)	101.9 ± 5.4	102.2 ± 5.3	-1.00 ± 1.88**	-0.82 ± 2.29**	-0.18 (-0.69;0.32)	-0.09
TC^a ^(cm)	56.8 ± 3.6	57.0 ± 3.3	-0.77 ± 1.54**	-0.67 ± 2.05**	-0.08 (-0.54;0.36)	-0.06
SagD^a ^(cm)	21.8 ± 2.7	22.3 ± 3.0	-0.52 ± 1.50**	-0.83 ± 2.06**	0.22 (-0.19;0.62)	0.17
CC^a ^(cm)	38.8 ± 2.7	39.1 ± 2.3	-0.43 ± 0.88**	-0.41 ± 0.96**	-0.04 (-0.27;0.20)	-0.02
AC^a ^(cm)	32.6 ± 2.6	32.8 ± 2.5	-0.47 ± 1.22**	-0.42 ± 1.13**	-0.06 (-0.33;0.21)	-0.04
SBP^a ^(mmHg)	142.7 ± 16.8	145.6 ± 17.9	-4.19 ± 12.03**	-4.57 ± 14.68*	-0.29 (-3.24;2.69)	0.03
DBP^a ^(mmHg)	86.1 ± 10.1	86.1 ± 8.9	-2.89 ± 7.86**	-2.54 ± 7.21**	-0.38 (-1.98;1.21)	-0.05
TBF^a ^(%)	30.4 ± 4.5	30.6 ± 4.7	0.08 ± 2.49	-0.26 ± 2.80	0.32 (-0.30;0.94)	0.13

^a^WC = waist circumference, BW = body weight, BMI = Body Mass Index, HC = hip circumference, TC = thigh circumference, SagD = sagittal diameter, CC = calf circumference. AC = arm circumference, SBP = systolic blood pressure, DBP = diastolic blood pressure, TBF = total body fat. Data are presented as mean ± standard deviation unless stated otherwise. *p < 0.05 and *p < 0.0001 for within-group-changes. ^† ^Estimates for treatment effect (95% confidence interval) reflect the between-group-difference and are adjusted for baseline measurements and cluster randomisation. ^‡ ^Cohen's *d*. Small: *d *≤ 0.33; Medium: 0.33 <*d *< 0.55; Large: *d *≥ 0.56.

### Immediate outcomes: effects after the intervention period

At 12-months follow-up, there was a significant decline (mean change) in waist circumference (INT: -2.32 cm; CON: -1.9 cm) and body weight (INT: -1.86 kg; CON: -1.62 kg). Although the declines were greater in the intervention group, the between-group-differences were not significant (p = 0.16 and p = 0.43, respectively) (Table 2). Similarly, systolic blood pressure was reduced in both the intervention (-6.50 mmHg) and control (-4.59 mmHg) group in men. The reduction was significantly greater (p = 0.05) in the intervention group, whereas the reduction in diastolic blood pressure did not differ across the groups (INT: -4.03 mmHg; CON: -2.79 mmHg; p = 0.08).

Among men in both groups daily activities (INT: 62.5 min/week; CON: 80.7 min/week), sum of household activities (INT: 0.9; CON: 1.0), and total physical activity ((PASE) INT: 22.9; CON: 17.6) increased significantly. However, the between-group-differences were not significant (p = 0.98, p = 0.47, p = 0.81 respectively) (Table 3). Men in the intervention group increased their fruit and vegetable consumption, and decreased their intake of sliced meat, meat, sugar added to tea, fat intake and total energy intake significantly. Men in the control group however also decreased their meat and sugar in tea consumption, and total energy intake. None of the between-group-differences was significant (Table 3).

**Table 3:** Change and treatment effect for physical activity and dietary intake in men after A) 12 and B) 24 months follow-up

A)	Baseline		Follow-up at 12 months			
			Change		Treatment effect^†^	*d*^‡^
	intervention	control	intervention	control		
Number	174	178	164	170		
Daily routine PA^a ^(min/wk)	286 ± 264	244 ± 212	62.5 ± 287.9*	80.7 ± 281.7*	-0.68 (-57.7;56.3)	-0.06
Recreation/sports PA^a ^(min/wk)	270 ± 423	250 ± 321	50.9 ± 463.5	38.6 ± 362.6	24.26 (-40.3;88.9)	0.03
Σ household activities (0-6)	2.5 ± 1.3	2.4 ± 1.4	0.9 ± 1.3**	1 ± 1.5**	-0.1 (-0.4;0.2)	-0.07
PASE^a^	116.3 ± 64.4	122.4 ± 68.3	22.9 ± 64.4**	17.6 ± 69.9*	1.35 (-9.84;12.5)	0.08
Fruit & vegetable (g/MJ)	31.2 ± 16.8	32 ± 16.4	4.4 ± 22.4*	2.2 ± 17.2	1.68 (-2.7;6.1)	0.11
Fat intake (en%)	34.5 ± 5.4	33.7 ± 5.2	-1.4 ± 5.5*	-0.4 ± 4.8	-0.7 (-1.7;0.3)	-0.19
Energy intake (MJ/day)	9.5 ± 2.1	9.8 ± 2.2	-1 ± 1.9**	-0.8 ± 1.9**	-0.24 (-0.6;0.1)	-0.12
Sliced meat (serving/month)	39.7 ± 28.1	39.7 ± 29.9	-4.7 ± 27.3*	-2.4 ± 26.4	-2.61 (-8.0;2.8)	-0.09
Meat (serving/month)	20.5 ± 9.2	20.9 ± 10.2	-2.8 ± 8.6**	-1.9 ± 10.5*	-1 (-2.8;0.9)	-0.09
Beer (serving/month)	23.1 ± 26.5	29.4 ± 30.4	0.2 ± 21.3	-2 ± 20.6	1.01 (-4.0;6.0)	0.11
Wine (serving/month)	26 ± 24.9	24.7 ± 23.4	-0.8 ± 17.5	-0.3 ± 15.5	-0.47 (-4.5;3.6)	-0.03
Sugar in tea (cubes/cup)	0.5 ± 0.6	0.6 ± 0.6	-0.2 ± 0.6**	-0.3 ± 0.6**	0.08 (-0.03;0.2)	0.18
Sugar in coffee (cubes/cup)	0.5 ± 0.8	0.5 ± 0.7	-0.1 ± 0.5	-0.1 ± 0.7	0 (-0.1;0.1)	-0.05
Milk in coffee (cubes/cup)	0.6 ± 0.6	0.5 ± 0.6	0 ± 0.9	0.1 ± 0.9	-0.08 (-2.0;0.1)	-0.10
						
**B)**	**Baseline**		**Follow-up at 24 months**			
			**Change**		**Treatment effect**^†^	***d*^‡^**
	**intervention**	**control**	**intervention**	**control**		

Number	174	178	153	155		
Daily routine PA^a ^(min/wk)	286 ± 264	244 ± 212	92.8 ± 354.6*	122.6 ± 273.2**	-9.92 (-73.6;53.8)	-0.09
Recreation/sport PA^a ^(min/wk)	270 ± 423	250 ± 321	74.5 ± 499.6	23.4 ± 310.7	70.24 (5.6;134.9)	0.12
Σ household activities (0-6)	2.5 ± 1.3	2.4 ± 1.4	0.9 ± 1.3**	1.2 ± 1.5**	-0.29 (-0.5;0.0)	-0.19
PASE^a^	116.3 ± 64.4	122.4 ± 68.3	22.6 ± 67.5**	19.0 ± 64.9*	-1.27 (-11.6;9.7)	0.05
Fruit & vegetable (g/MJ)	31.2 ± 16.8	32 ± 16.4	4.6 ± 17.9*	0.5 ± 17	2.99 (-0.5;6.5)	0.24
Fat intake (en%)	34.5 ± 5.4	33.7 ± 5.2	-1 ± 5.7*	0.1 ± 4.9	-0.5 (-1.5;0.5)	-0.20
Energy intake (MJ/day)	9.5 ± 2.1	9.8 ± 2.2	-0.8 ± 1.9**	-0.8 ± 2.2**	-0.04 (-0.5;0.4)	-0.01
Sliced meat (serving/month)	39.7 ± 28.1	39.7 ± 29.9	-3.6 ± 28.4	-6.1 ± 26.6*	1.88 (-3.2;7.0)	0.09
Meat (serving/month)	20.5 ± 9.2	20.9 ± 10.2	-1.5 ± 9.3*	-1.3 ± 8.8	-0.36 (-2.3;1.6)	-0.03
Beer (serving/month)	23.1 ± 26.5	29.4 ± 30.4	-1.8 ± 19.9	-3.7 ± 23	-0.52 (-5.4;4.4)	0.09
Wine (serving/month)	26 ± 24.9	24.7 ± 23.4	-0.3 ± 20.5	0.7 ± 18.6	-0.60 (-4.6;3.4)	-0.05
Sugar in tea (cubes/cup)	0.5 ± 0.6	0.6 ± 0.6	-0.3 ± 0.6**	-0.3 ± 0.7**	0 (-0.1;0.1)	0.02
Sugar in coffee (cubes/cup)	0.5 ± 0.8	0.5 ± 0.7	-0.1 ± 0.5*	-0.1 ± 0.7	0 (-0.1;0.1)	-0.06
Milk in coffee (cubes/cup)	0.6 ± 0.6	0.5 ± 0.6	0 ± 0.5	0.0 ± 0.4	-0.02 (-0.1;0.1)	-0.08

^a^Physical Activity (Scale for the Elderly). Data are presented as mean ± standard deviation unless stated otherwise. *p < 0.05 and *p < 0.0001 for within-group-changes. ^† ^Estimates for treatment effect (95% confidence interval) reflect the between-group-difference and are adjusted for baseline measurements and cluster randomisation. ^‡ ^Cohen's *d*. Small: *d *= 0.33; Medium: 0.33 <*d *< 0.55; Large: *d *= 0.56.

### Long term outcomes: effects one year after cessation of the intervention

Waist circumference in men in the intervention and control group remained lower compared to baseline, though during the second year of follow-up an increase was observed. Body weight stabilised in both groups. Again the between-group-differences for waist circumference and body weight were not statistically significant (Table 2).

Change in sport and recreational activities was higher after two year in the intervention group (74.5 min/week) compared to the control group (23.4 min/week; p = 0.03). The sum of household activities increased in both groups (INT: 0.9; CON: 1.2; p = 0.03) in favour of the control group. For other lifestyle behaviours mentioned in Table 3 none of the between-group-differences were statistically significant.

### Secondary analyses

Secondary analyses among men with low educational level (INT n = 44; CON n = 41) revealed a significant between-group-difference in waist circumference of -1.56 cm (95% CI: -2.91 to -0.21; p = 0.03) at 12 month follow-up in favour of the intervention group. In this subgroup the reduction in body weight was also larger in the intervention group compared to the control group. However, the between-group-difference of -0.96 kg (95%CI: -2.40 to 0.47) was not significant. There were no significant differences in changes in waist circumference and body weight between the intervention and control group observed in any other predefined subgroup.

Among men with a low educational level, also fat intake decreased (mean ± SD) (-3.3 ± 6.5 en%; n = 41) in the intervention group, while an increase was observed in the control group (0.6 ± 4.2 en%; n = 39). The between-group-difference was -3.2 en% (p = 0.01).

The per protocol analyses to test the influence of utilisation of the key modules did not reveal any differences between those that had actually utilised the modules once or more versus the randomly selected sex-matched control group at 12 and 24-months follow-up (data not shown).

## Discussion

Retired subjects participating in a one- year low intensity energy balance programme decreased their waist circumference, body weight, BMI, blood pressure and most other body composition indices and improved their physical activity and dietary behaviour. Although the changes were more consistent and more pronounced among subjects of the intervention group, the between-group-differences were small and mostly not statistically significant. Additional analyses among low educated men indicate that the programme may be effective in men with a low educational level: for waist circumference and fat intake the between-group-differences were significantly different. After the follow-up period the between-group-differences more or less remained the same, though the magnitude of the differences diminished.

We hypothesised that the intervention group would maintain their waist circumference and body weight in the two years following transition to retirement as opposed to the control group. Within the control group, the waist circumference would on average increase by 0.5 cm per year. Remarkably, in men, both groups reduced their body weight and weight circumference. And although the difference in change was -0.48, we could not demonstrate a significant effect. Possibly our study lacked statistical power. The sample size was calculated based on observational data on change in waist circumference in a middle-aged population. Such data were not available for the specific group of retirees we studied. Apparently, the variance that was used for the power calculation was too low. Further, despite the randomisation, the control group had on average higher, though non significant, scores for the outcome measurements at baseline, which may have caused regression to the mean. However, we included baseline values in the models and thus have allowed for these apparent differences.

The lack of effect may also be due to our recruitment strategy resulting in a relatively healthy and health conscious group of subjects. The study participants were selected from pre-retirement workshops, often attended by higher socio-economic groups, who in general are more motivated to change physical activity and diet, which might have reduced the added value of the prevention programme. Earlier studies have described that individuals willing to participate in health promoting intervention studies are already interested in diet and physical activity and are health conscious [30,31].

Further, study participation itself may have led to increased awareness and motivation to change physical activity and/or diet in the control group and intervention group (Hawthorne effect), which also reduced the added value of the programme. The influence of the researchers or others involved in measurements is supposed to be very low, since information associated with the content of the intervention was not discussed at the physical examinations or during other contacts.

And last but not least, transition to occupational retirement per se may have induced the changes in (lifestyle) behaviours. The study by Nooyens *et al*, showed that in transition to retirement subjects decrease work-related activity and increase household activities as well as doing odd jobs [9]. Maybe the impact of retirement itself was so great resulting in either a ceiling effect or a small added value of the intervention programme.

A limitation of the study is the small number of females. Although the percentage of women that participated is representative for the percentage of women that worked in this age group, the number was too small to draw conclusions on the effectiveness of the intervention. And although the PASE questionnaire was originally designed for older adults (65+ years) it was chosen because it distinguishes activities (household -and leisure activities) that are relevant for retired people. Moreover, the recall period was only one week which also enabled us to pick up changes over a short period of time.

The programme of our study was developed according to the Intervention Mapping Protocol [15]. This systematic process comprises a series of five steps for the development of health promotion programmes based on theory, empirical evidence, and additional research [7]. Since information on determinants of weight gain for the specific group of retirees or middle-aged adults was lacking we used available information from general adult populations. It subsequently appeared to be a good choice, since Nooyens *et al *showed that determinants of weight gain among older populations do not really differ from determinants in adult populations [9].

Our programme aimed to induce relatively small and possibly sustainable changes in physical activity and diet to prevent weight gain [7,10].

Moreover, the programme was developed in a way that it could be implemented nationwide, thus it was of low intensity, easily accessible and home-based. As a result, participants could voluntarily use the modules of the programme in accordance with their personal preferences. As a consequence commitment and adherence of the target group may have been too weak to result in a behaviour change.

Although the use of personal computers and internet in the middle-aged has increased rapidly in recent years [32], it is unknown to what extent this is a suitable mode to deliver health messages to this age group, as can be concluded from the data on utilisation. Only 47% of the participants in the intervention group reported to have utilised both modules 2 and 3 (CD-ROMS with computer-tailored feedback), while the use of the Internet modules (study website and Weight Co@ch) was even lower: only 16% utilised Weight Co@ch. This indicates that the exposure to the full programme was rather low. Still, computer-tailored interventions have the potential to provide individualised behaviour change information to many individuals at low costs. This approach has been shown to be more effective than general nutrition information, especially for reduction of dietary fat intake [33], although effect sizes are mostly small and apply only to the short and medium term (follow-up up to six months) [34]. Clearly, the delivery of computer tailored interventions in real-life settings needs more research [35].

The results of this study can by used by the Netherlands Heart Foundation and others to further improve the intervention modules. At present it is not clear if or how the results of this study will lead to further development or implementation of this intervention.

## Conclusions

The individually tailored one year energy balance programme did not have a significant effect on any of the outcomes in recent retirees though it showed a pattern of small, non- significant effects on changes in body composition, physical activity and dietary behaviour. Lack of power may partly account for these findings. Apparently transition to occupational retirement and/or participation in research had a greater impact than the intervention programme itself.

## Competing interests

The authors declare that they have no competing interests.

## Authors' contributions

AW, AJS, FJK and EGS were the principal investigators of the study and developed the concept and the design of the study. PJMH contributed to the body composition assessment, AS and SK contributed to the behavioural parts of the intervention and to the assessment of behaviour. AW performed the analyses and AW and JS drafted the manuscript. All authors read and approved the final manuscript.

## Pre-publication history

The pre-publication history for this paper can be accessed here:

http://www.biomedcentral.com/1471-2458/10/110/prepub
